# Endoscopic screening using esophageal iodine staining and genotypes of ADH1B and ALDH2 in Japanese alcohol-dependent women

**DOI:** 10.1371/journal.pone.0210546

**Published:** 2019-01-10

**Authors:** Akira Yokoyama, Tetsuji Yokoyama, Tai Omori, Hitoshi Maesato, Tsuyoshi Takimura, Chie Iwahara, Mitsuru Kimura, Toshifumi Matsui, Takeshi Mizukami, Katsuya Maruyama

**Affiliations:** 1 National Hospital Organization Kurihama Medical and Addiction Center, Yokosuka, Kanagawa, Japan; 2 Department of Health Promotion, National Institute of Public Health, Wako, Saitama, Japan; 3 Endoscopy Center, Kawasaki Municipal Ida Hospital, Kawasaki, Kanagawa, Japan; Yale University School of Medicine, UNITED STATES

## Abstract

**Background:**

The presence of large or multiple esophageal distinct iodine-unstained lesions (DIULs) is a strong predictor of field cancerization in the upper aerodigestive tract. Several risk factors for DIULs, including genetic polymorphisms of alcohol and aldehyde dehydrogenases (ADH1B, rs1229984; ALDH2, rs671), have been demonstrated in Japanese alcohol-dependent men. However, few evaluations of alcohol-dependent women have been conducted in this field.

**Methods:**

Using multiple logistic regression models, we investigated the results of screening using esophageal iodine staining and the identification of determinants for esophageal DIULs in 472 Japanese alcohol-dependent women.

**Results:**

DIULs ≥5 mm, multiple DILUs, and both characteristics were observed in 35 (7.4%), 31 (6.6%), and 16 (3.4%) patients, respectively. DIULs ≥5 mm were histologically diagnosed as low-grade intraepithelial neoplasia in 26 patients and superficial squamous cell carcinoma in 9 patients. Although the inactive heterozygous ALDH2*1/*2 genotype was more common (33.3% vs. 11.4%, p = 0.002) in the group with DIULs ≥5 mm than in the group without DIULs ≥5 mm, no significant differences in the results of a questionnaire asking about current and past facial flushing after a glass of beer were seen between the groups with and without DIULs ≥5 mm. When individuals with current or former flushing were assumed to have inactive ALDH2, the sensitivity and specificity of current or former flushing to identify the presence of inactive ALDH2 were 50.0% and 93.5%, respectively; these values were previously reported to be 88% and 92%, respectively, in a Japanese general female population. The low sensitivity in the present study suggests that a lack of alcohol flushing may play a crucial role in the development of alcohol dependence in women with inactive ALDH2. No significant differences in age, usual alcohol consumption, or smoking habits were observed according to ADH1B and ALDH2 genotypes. Multiple logistic regression analyses showed that the slow-metabolizing ADH1B*1/*1 genotype (odds ratio [95% confidence interval], 12.5 [4.82–32.4] and 9.89 [3.50–27.9]), the inactive heterozygous ALDH2*1/*2 genotype (2.94 [1.18–7.38] and 3.79 [1.40–10.3]), a lower body mass index per -1 kg/m2 (1.17 [1.02–1.35] and 1.38 [1.14–1.67]), and a mean corpuscular volume ≥106 fl (3.70 [1.56–8.81] and 3.27 [1.24–8.64]) increased the risk of DIULs ≥5 mm and multiple DIULs, respectively. The combination of ADH1B*1/*1 and ALDH2*1/*2 markedly increased the risk of esophageal DIULs ≥5 mm (39.3 [10.6–146]).

**Conclusions:**

Japanese alcohol-dependent women shared several common risk factors for esophageal squamous cell neoplasia with alcohol-dependent men, but with considerably different magnitudes.

## Introduction

Genetic polymorphisms of alcohol dehydrogenase-1B (ADH1B; rs1229984) and aldehyde dehydrogenase-2 (ALDH2; rs671) modulate exposure levels to ethanol and acetaldehyde, two established human carcinogens [1], after drinking alcohol. The risks of squamous cell carcinoma (SCC) [2–7] and low-grade intraepithelial neoplasia (LGIN) [8–10], especially of multiple SCC and multiple LGIN [2,7–12], in the esophagus are reportedly increased by the presence of the slow-metabolizing ADH1B*1/*1 genotype and the inactive heterozygous ALDH2*1/*2 genotype in East Asian drinkers. These genetic polymorphisms have been investigated mainly in relation to alcohol metabolism and alcohol drinking behaviors [13], but a recent phenome-wide association study has highlighted that *ADH1B* rs1229984 is associated with a wide range of phenotype traits including psychological traits and socioeconomic status, some of which do not appear to be mediated by alcohol [14].

However, much of the cancer research in this field has focused on male drinkers, since women account for a relatively small proportion of esophageal SCC patients among East Asians. The mortality rate for esophageal cancer per 100,000 Japanese women in 2012 was estimated to be 2.9, whereas that for Japanese men was 15.9 [15]. Gender differences in drinking and smoking habits explain much of this difference. According to the National Health and Nutrition Survey conducted in Japan in 2012 [16], the prevalence of current smokers and the proportion of habitual drinkers who drink on 3 or more days/week and consume at least 22 g of ethanol each time were 34.1% and 34.0%, respectively, in male adults, but only 9.0% and 7.3%, respectively, in female adults.

However, a case-control study in Japanese women showed a markedly high risk of esophageal SCC in women with the ALDH2*1/*2 genotype who were heavy drinkers [17]; this result was comparable to the results of a male study conducted simultaneously [3]. A Japanese genome-wide association study also showed similar odds ratios (ORs) for esophageal SCC for the ADH1B*1/*1 genotype and the ALDH2*1/*2 genotype between men and women [5]. Meta-analyses of Asian case-control studies showed that female drinkers with the ALDH2*1/*2 genotype had an increased risk of esophageal SCC [18,19]. However, results regarding the effects of the ADH1B*1/*1 genotype and gender differences are conflicting probably due to the small number of studies, and the small proportion of heavy drinkers and ADH1B*1/*1 carriers in Asian female populations [17–20].

The present study was conducted in Japanese alcohol-dependent women who underwent screening involving a combination of endoscopy and esophageal iodine staining; we evaluated whether and to what extent the ALDH2*1/*2 genotype, the ADH1B*1/*1 genotype, smoking, and other factors were associated with esophageal neoplasia.

## Materials and methods

At the Kurihama Medical and Addiction Center, we have been providing a cancer-screening program for alcohol-dependent women that consists of endoscopy combined with esophageal iodine staining. The screening program and diagnostic procedure used in the present study were described in a previous report [9]. The proposal for this study was reviewed and approved by the Kurihama Medical and Addiction Center Institutional Review Board, and written informed consent was obtained from all the participating patients.

### Patients

The patients were 472 Japanese alcohol-dependent women between the ages of 30 and 79 years who had visited the Center for the treatment of alcohol dependence, had no history of cancer in the esophagus or the head and neck region, and had completed the initial cancer screening examination, including esophageal iodine staining, between 2006 and 2017. Patients who participated in the alcoholism-treatment program were given an explanation of the usefulness of iodine-staining screening for the early detection of esophageal SCC and the high detection rate, and were given the opportunity to undergo screening. The Center used the DSM-IV criteria for alcohol dependence [21] between 2006 and 2010 and the ICD-10 criteria for alcohol dependence [22] between 2011 and 2016. All the patients met the criteria for alcohol dependence. Prior to the endoscopic examination, the endoscopists asked the patient about her alcohol consumption during the preceding year and smoking habit using a structured questionnaire as described in detail previously [9]. On the day of each patient’s first visit to the Center, her body height was measured after removing her shoes, and she was weighed while wearing light clothing.

### Endoscopic procedure

**Fig 1:**
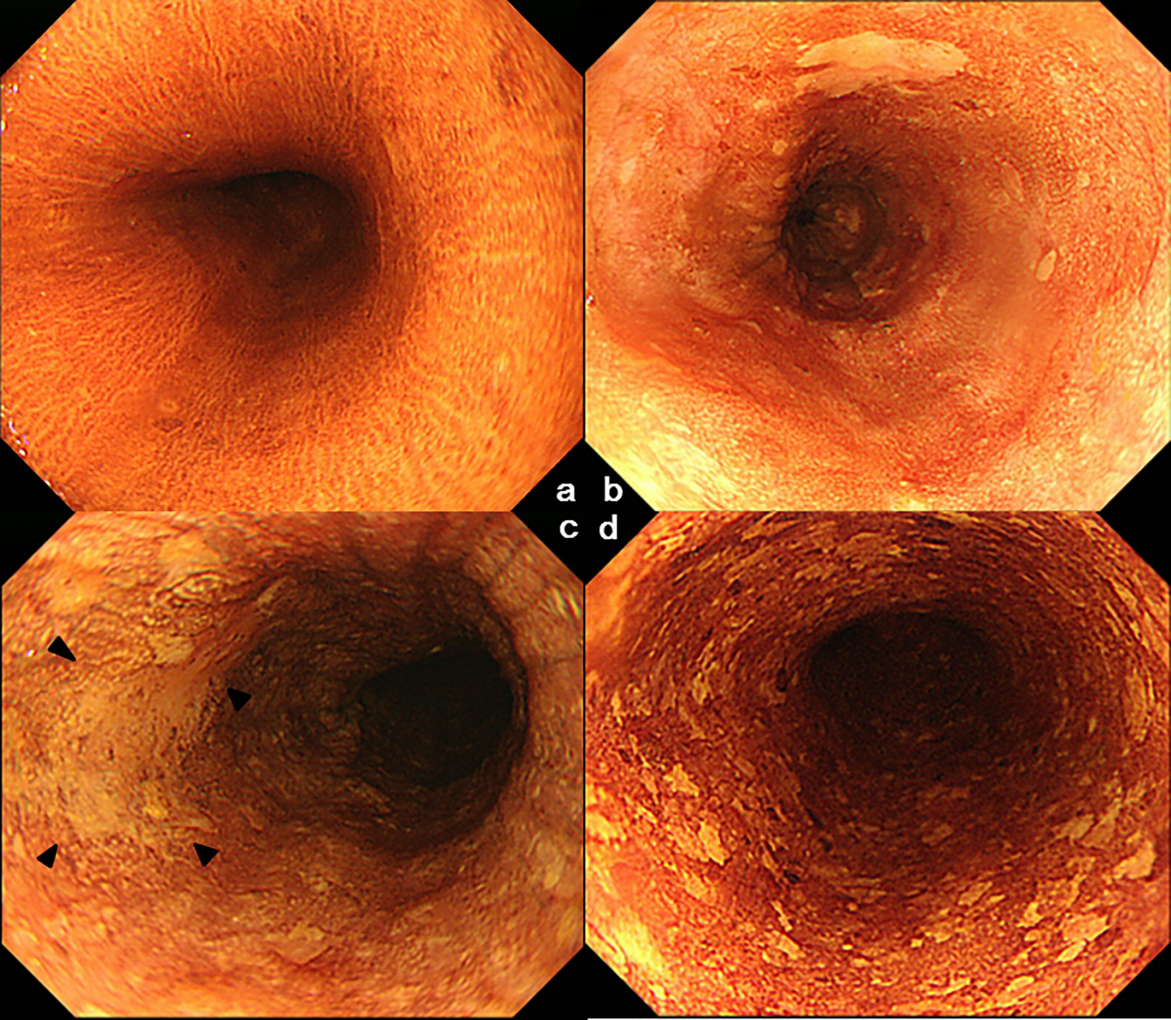
No distinct iodine-unstained lesions (DIULs) ≥5 mm (a). The DIUL ≥5 mm was diagnosed as LGIN (b) and as superficial SCC (c). ‘‘Multiple DIULs” was defined as present when 10 or more DIULs of any size were observed in at least one endoscopic field of view (d).

The endoscopy examinations were performed with an Olympus XQ230, Q240, Q240Z, Q260, or Q260Z panendoscope (Olympus Optical Co. Ltd., Tokyo, Japan) as described in detail previously [9]. Mucosal biopsy specimens were collected from distinct iodine-unstained lesions (DIULs; Fig 1) whose greatest diameter was 5 mm or more using standard oval biopsy forceps with a needle (Olympus FB-34K-1). The presence of multiple DIULs was recorded when 10 or more DIULs of any size were observed in at least one endoscopic field of view.

### Histological diagnosis of DIULs ≥5 mm

Each DIUL was histologically diagnosed according to the degree of cytologic and architectural atypia [23]. When the lesions were resected by endoscopic mucosectomy or radical surgery, the histological diagnosis of the resected specimen was regarded as the final diagnosis.

### Mean corpuscular volume

During each patient’s initial visit to the Center for the treatment of alcohol dependence, we measured the mean corpuscular volume (MCV) using the electrical impedance method with an autoanalyzer (CELL-DYN 3500; Abbott, North Chicago, IL). We dichotomized the results into an MCV <106 fl group and an MCV ≥106 fl group, since macrocytosis with an MCV value ≥106 fl was found to be associated with an increased risk of SCC in the upper aerodigestive tract in our previous studies in alcohol-dependent men [24,25].

### Flushing questionnaire

We interviewed the patients with regard to flushing by asking two simple questions just prior to the endoscopic screening as described previously [26]: (a) “Do you have a tendency to flush in the face immediately after drinking a glass of beer (yes, no, or unknown)?” and (b) “Did you have a tendency to flush in the face immediately after drinking a glass of beer during the first 1–2 years after you started drinking (yes, no, or unknown)?” Individuals who answered “yes” to question (a) were recorded as “current flushing,” while those who answered “no” to question (a) and “yes” to question (b) were recorded as “former flushing.” The remaining subjects were recorded as “never flushing.”

### ALDH2 and ADH1B genotyping

We determined the ALDH2 and ADH1B genotypes of all the 401 patients from whom informed consent was obtained on admission to the Center. PCR-restriction fragment length polymorphism methods were used to genotype ALDH2 and ADH1B in DNA samples obtained from blood as described in detail previously [2].

### Statistical analysis

Values were expressed as the mean and standard deviation (SD) or the percentage. *P* values for categorical data were calculated using Fisher’s exact test or the Cochran-Mantel-Haenszel test for homogeneity or trend, where appropriate. Student’s *t*-test was used to compare the mean values between two groups, and the general linear model was used to test for trends. The multivariate odds ratios (ORs) and the 95% confidence intervals (CIs) were calculated using multiple logistic models. The confounding factors that were added to the models were age, usual alcohol consumption, daily number of cigarettes currently smoked, body mass index (BMI), MCV, and ADH1B and ALDH2 genotypes. We combined the ADH1B*1/*2 genotype carriers and the ADH1B*2/*2 genotype carriers into a group with the ADH1B*2 allele because of the a semi-dominant nature of the ADH1B*2 allele [27,28]. *P* values were adjusted for multiple testing by Bonferroni-Holm’s method where appropriate. Statistical significance was defined as a *P* value of <0.05. All the statistical analyses were performed using the SAS software program (version 9.4; SAS Institute, Cary, NC).

## Results

DIULs ≥5 mm were observed in 35 (7.4%) of the 472 patients. The DIUL was diagnosed as LGIN (mild or moderate dysplasia) in 26 (5.5%) patients and as superficial SCC in 9 (1.9%). The esophageal SCC was confined to the epithelium in 6 patients and had invaded the proper mucosal layer in two patients and the muscularis mucosae in one patient.

The basic characteristics of the patients according to whether they had squamous cell neoplasia (LGIN or SCC), as diagnosed by the presence of DIULs ≥5 mm, are shown in Table 1. A significantly older age, smaller BMI, and larger MCV were observed in the groups with neoplasia. The slow-metabolizing ADH1B*1/*1 genotype and the inactive heterozygous ALDH2*1/*2 genotype were significantly more common in the groups with neoplasia. In the control group without the DIULs ≥5 mm the frequency of ADH1B genotype was significantly deviated from Hardy-Weinberg equilibrium (p<0.0001) overrepresenting *1 allele, and the frequency of ALDH2 genotype was not significantly deviated from Hardy-Weinberg equilibrium (p = 0.25).

**Table 1:** Background characteristics of Japanese alcohol-dependent women according to the results of endoscopic screening.

	Total	Esophageal DIULs ≥5 mm					
		Absence	Presence	*P* ^†^	LGIN	SCC	*P* ^‡^
n (%)	472	437 (92.6)	35 (7.4)		26 (5.5)	9 (1.9)	
Age (years old) (n,%)							
30–39	110	106 (96.4)	4 (3.6)	*0*.*020*	2 (1.8)	2 (1.8)	0.068
40–49	169	157 (92.9)	12 (7.1)		8 (4.7)	4 (2.4)	
50–59	118	109 (92.4)	9 (7.6)		8 (6.8)	1 (0.8)	
60–69	60	52 (86.7)	8 (13.3)		7 (11.7)	1 (1.7)	
70–79	15	13 (86.7)	2 (13.3)		1 (6.7)	1 (6.7)	
Mean±SD	48.2±10.6	47.9±10.6	52.1±10.7	*0*.*025*	53.3±9.8	48.6±13.0	0.085
Alcohol consumption (g ethanol/day)							
Mean±SD	131.4±84.5	132.3±86.7	120.1±48.4	0.19	117.8±49.7	127.0±46.7	0.50
Smoking (cigarettes/day) (n, %)							
Never-smoker	134	125 (93.3)	9 (6.7)	0.54	6 (4.5)	3 (2.2)	0.94
Ex−smoker	37	34 (91.9)	3 (8.1)		2 (5.4)	1 (2.7)	
1–19	147	139 (94.6)	8 (5.4)		4 (2.7)	4 (2.7)	
20-	154	139 (90.3)	15 (9.7)		14 (9.1)	1 (0.6)	
Mean±SD	11.4±11.7	11.3±11.7	12.6±11.4	*0*.*51*	14.7±11.7	6.6±8.3	0.99
Body Mass Index (kg/m^2^) ^§^							
<18.5	130	116 (89.2)	14 (10.8)	0.037	10 (7.7)	4 (3.1)	0.043
18.5–22.0	195	182 (93.3)	13 (6.7)		9 (4.6)	4 (2.1)	
22.1–24.9	83	77 (92.8)	6 (7.2)		6 (7.2)	0 (0.0)	
25.0+	61	60 (98.4)	1 (1.6)		0 (0.0)	1 (1.6)	
Mean±SD	20.8±3.8	20.9±3.8	19.4±2.9	0.006	19.4±3.0	19.3±3.0	0.032
MCV (fl) (n, %)							
<106	344	326 (94.8)	18 (5.2)	*0*.*005*	14 (4.1)	4 (1.2)	0.003
≥106	128	111 (86.7)	17 (13.3)		12 (9.4)	5 (3.9)	
Mean±SD	99.9±10.1	99.6±10.1	103.8±8.1	0.006	103.6±8.2	104.4±8.5	0.021
*ADH1B* genotype (n, %)^§^							
*1/*1	126	102 (81.0)	24 (19.0)	< .0001	17 (13.5)	7 (5.6)	< .0001
*1/*2	133	128 (96.2)	5 (3.8)		3 (2.3)	2 (1.5)	
*2/*2	142	138 (97.2)	4 (2.8)		4 (2.8)	0 (0.0)	
*ALDH2* genotype (n, %)^§^							
*1/*1	348	326 (93.7)	22 (6.3)	0.002	19 (5.5)	3 (0.9)	< .0001
*1/*2	53	42 (79.2)	11 (20.8)		5 (9.4)	6 (11.3)	
Alcohol flushing (n, %)^§^							
Never flushing	398	369 (92.7%)	29 (7.3%)		22 (5.5%)	7 (1.8%)	
Current flushing	44	42 (95.5%)	2 (4.5%)		1 (2.3%)	1 (2.3%)	
Former flushing	15	13 (86.7%)	2 (13.3%)	0.79	1 (6.7%)	1 (6.7%)	0.51
Multiple DIULs of any size							
Absent	441	422 (95.7%)	19 (4.3%)		15 (3.4%)	4 (0.9%)	
Present	31	15 (48.4%)	16 (51.6%)	< .0001	11 (35.5%)	5 (16.1%)	< .0001

ADH1B, alcohol dehydrogenase-1B; ALDH2, aldehyde dehydrogenase-2; DIULs, distinct iodine-unstained lesions; MCV, mean corpuscular volume; LGIN, low grade intraepithelial neoplasia; SCC, squamous cell carcinoma

†Comparison between absence and presence. *P* values for categorical data are using the Fisher's exact test for MCV and ALDH2, and the Cochran-Mantel-Haenszel test for homogeneity for smoking, and for trend for other variables; *P* values for mean values are using the *t*-test.

‡Comparison of absence, LGIN, and SCC. *P* values for categorical data are using the Cochran-Mantel-Haenszel test for homogeneity for smoking, and for trend for other variables; *P* values for mean values are for trend using a general linear model.

§ Due to missing values, the numbers do not add up to 472.

No significant differences in usual alcohol consumption, daily number of cigarettes currently smoked, and the results of the simple flushing questionnaire were seen between the groups. Multiple DIULs of any size were observed in 31 (6.6%) patients. The prevalence of multiple DIULs was 3.4% among the patients without DIULs ≥5 mm, as opposed to 45.7%, 42.3%, and 55.6% among the patients with DIULs ≥5 mm, LGIN, and SCC, respectively.

No significant differences in age, usual alcohol consumption, or smoking habits were seen according to the ADH1B and ALDH2 genotypes (Table 2). No significant differences in BMI or MCV were seen according to the ADH1B genotype. However, a significantly smaller BMI and a larger MCV were observed in the ALDH2*1/*2 group, compared with the ALDH2*1/*1 group.

**Table 2:** Background characteristics of Japanese alcohol-dependent women according to ADH1B and ALDH2 genotypes.

	*ADH1B*				*ALDH2*		
	*1/*1	*1/*2	*2/*2	*P* ^†^	*1/*1	*1/*2	*P*
n (%)	126	133	142		348	53	
Age (years old) (n,%)							
30–39	21 (24.7)	29 (34.1)	35 (41.2)	0.37	72 (84.7)	13 (15.3)	0.36
40–49	50 (33.8)	49 (33.1)	49 (33.1)		127 (85.8)	21 (14.2)	
50–59	33 (33.0)	30 (30.0)	37 (37.0)		86 (86.0)	14 (14.0)	
60–69	19 (34.5)	21 (38.2)	15 (27.3)		51 (92.7)	4 (7.3)	
70–79	3 (23.1)	4 (30.8)	6 (46.2)		12 (92.3)	1 (7.7)	
Mean±SD	49.3±10.2	48.6±10.7	47.9±10.9	0.36	48.8±10.8	47.0±9.0	0.36
Alcohol consumption (g ethanol/day)							
Mean±SD	131.9±78.6	138.2±95.5	128.1±80.7	0.92	133.6±88.0	126.2±63.9	0.92
Smoking (cigarettes/day) (n, %)							
Never-smoker	42 (36.5)	33 (28.7)	40 (34.8)	0.84	102 (88.7)	13 (11.3)	0.30
Ex-smoker	11 (31.4)	10 (28.6)	14 (40.0)		34 (97.1)	1 (2.9)	
1–19	34 (28.1)	45 (37.2)	42 (34.7)		104 (86.0)	17 (14.0)	
20-	39 (30.0)	45 (34.6)	46 (35.4)		108 (83.1)	22 (16.9)	
Mean±SD	10.2±11.3	11.9±11.9	11.5±12.0	0.38	10.8±11.4	14.5±13.2	0.11
Body Mass Index (kg/m^2^)							
<18.5	28 (25.5)	37 (33.6)	45 (40.9)	0.56	86 (78.2)	24 (21.8)	0.034
18.5–22.0	56 (32.7)	65 (38.0)	50 (29.2)		154 (90.1)	17 (9.9)	
22.1–24.9	26 (37.1)	20 (28.6)	24 (34.3)		64 (91.4)	6 (8.6)	
25.0+	16 (33.3)	10 (20.8)	22 (45.8)		43 (89.6)	5 (10.4)	
Mean±SD	21.3±3.7	20.2±3.0	20.8±4.2	0.37	20.9±3.7	19.7±3.5	0.058
MCV (fl) (n, %)							
<106	102 (34.2)	93 (31.2)	103 (34.6)	0.13	272 (91.3)	26 (8.7)	< .0001
≥106	24 (23.3)	40 (38.8)	39 (37.9)		76 (73.8)	27 (26.2)	
Mean±SD	99.1±8.3	101.1±8.6	99.9±10.7	0.52	99.1±8.7	106.1±11.0	< .0001

ADH1B, alcohol dehydrogenase-1B; ALDH2, aldehyde dehydrogenase-2; MCV, mean corpuscular volume

† Comparison of *1/*1, *1/*2, and *2/*2. *P* values for categorical data are using the Cochran-Mantel-Haenszel test for homogeneity for smoking, and for trend for other variables; *P* values for mean values are for trend using a general linear model. *P* values are adjusted for multiplicity (2 comparisons for each characteristic) by Bonferroni-Holm’s method.

When current or former flushing individuals were assumed to have inactive ALDH2, the sensitivity and specificity of current or former flushing to identify the presence of inactive ALDH2 were 50.0% and 93.5%, respectively (Table 3). Current or former flushing was reported less frequently among the ADH1B*1/*1 carriers than among the ADH1B*2 carriers regardless of the ALDH2 genotype: 1.0% vs. 8.9% in the ALDH2*1/*1 group (*p* = 0.007) and 31.8% vs. 63.3% in the ALDH2*1/*2 group (*p* = 0.048).

**Table 3:** Combinations of ALDH2 and ADH1B genotypes in relation to the results of the alcohol flushing questionnaire in Japanese alcohol-dependent women.

			Results of the simple flushing questionnaire		
*ALDH2*	*ADH1B*	n	Never flushing	Current or former flushing	*P* ^†^
*1/*1	Any	337	93.5%	6.5%	
*1/*1	*1/*1	100	99.0%	1.0%	} 0.013
*1/*1	*2 carrier	237	91.1%	8.9%	
*1/*2	Any	52	50.0%	50.0%	
*1/*2	*1/*1	22	68.2%	31.8%	} 0.048
*1/*2	*2 carrier	30	36.7%	63.3%	

ADH1B, alcohol dehydrogenase-1B; ALDH2, aldehyde dehydrogenase-2

† Fisher's exact test adjusted for multiplicity (2 comparisons) by Holm’s method.

*P* = 0.42 for interaction of ALDH2 and ADH1B genotypes on alcohol flushing by a logistic regression analysis.

Determinants of esophageal DIULs ≥5 mm, LGIN, and SCC, and neoplasia were identified using a multiple logistic regression analysis (Table 4). The presence of the ADH1B*1/*1 genotype increased the odds ratios for DIULs ≥5 mm (OR [95% CI], 12.5 [4.82–32.4]), LGIN (11.0 [3.87–31.2]), and SCC (21.2 [2.51–180]), compared with the presence of the ADH1B*2 allele. The presence of the ALDH2*1/*2 genotype increased the OR for DIULs ≥5 mm (2.94 [1.18–7.38]) and SCC (18.2 [3.11–106]), compared with the presence of the ALDH2*1/*1 genotype. Other positive determinants were a younger age, a smaller BMI, and an MCV ≥106 fl for DIULs ≥5 mm.

**Table 4:** Multiple logistic analyses for identifying determinants of esophageal DIULs ≥5 mm in Japanese alcohol-dependent women.

	Esophageal DIULs ≥5 mm ^†^					
	Presence		LGIN		SCC	
	OR (95% CI)	*P*	OR (95% CI)	*P*	95% CI	*P*
Age, per +10 yrs	1.44 (0.96–2.15)	*0*.*080*	1.49 (0.94–2.38)	*0*.*092*	1.37 (0.60–3.11)	*0*.*46*
ADH1B *1/*1 vs. *2 carrier	12.5 (4.82–32.4)	*<* .*0001*	11.0 (3.87–31.2)	*<* .*0001*	21.2 (2.51–180)	*0*.*005*
ALDH2 *1/*2 vs. *1/*1	2.94 (1.18–7.38)	*0*.*021*	1.31 (0.40–4.24)	*0*.*65*	18.2 (3.11–106)	*0*.*001*
Alcohol consumption, per +22 g ethanol/day	1.01 (0.88–1.15)	*0*.*93*	0.98 (0.83–1.17)	*0*.*84*	1.11 (0.93–1.32)	*0*.*24*
Smoking, per +10 cigarettes/day	1.10 (0.77–1.55)	*0*.*61*	1.34 (0.92–1.94)	*0*.*13*	0.65 (0.28–1.49)	*0*.*31*
Body Mass Index, per -1 kg/m^2^	1.17 (1.02–1.35)	*0*.*030*	1.19 (1.01–1.40)	*0*.*043*	1.15 (0.90–1.47)	*0*.*28*
MCV, ≥106 fl vs. <106 fl	3.70 (1.56–8.81)	*0*.*003*	3.10 (1.18–8.11)	*0*.*022*	7.99 (1.32–48.4)	*0*.*024*

ADH1B, alcohol dehydrogenase-1B; ALDH2, aldehyde dehydrogenase-2; CI, confidence interval; DIULs, distinct iodine-unstained lesions; MCV, mean corpuscular volume; LGIN, low grade intraepithelial neoplasia; OR, odds ratio; SCC, squamous cell carcinoma

† Control: no DIULs ≥5 mm in the esophagus.

Multivariate odds ratios were estimated using a logistic regression model with all the variables entered.

Since the interaction of ALDH2 genotype by ADH1B genotype on the risk of esophageal DIULs ≥5 mm was not significant (see Table 5), the genetic variables were used in an independent manner.

Table 5 shows that the combination of ALDH2*1/*2 and ADH1B*1/*1 genotypes markedly increased the risk for DIULs ≥5 mm (39.3 [10.6–146]), LGIN (15.79 [3.36–74.11]), and SCC (249 [14.5->999]) for SCC.

**Table 5:** Multivariate odds ratios for combinations of ALDH2 and ADH1B genotypes in relation to esophageal DIULs ≥5 mm in Japanese alcohol-dependent women.

		Esophageal DIULs ≥5 mm						
		Absence (n = 368)	Presence (n = 33)		LGIN (n = 24)		SCC (n = 9)	
		n	n	OR (95% CI)	n	OR (95% CI)	n	OR (95% CI)
*ALDH2*	*ADH1B*							
*1/*1	*2 carrier	237	7	1 (ref.)	6	1 (ref.)	1	1 (ref.)
*1/*1	*1/*1	89	15	9.51 (3.33–27.1)	13	9.57 (3.11–29.44)	2	8.83 (0.63–124)
*1/*2	*2 carrier	29	2	1.43 (0.26–8.03)	1	0.78 (0.08–7.44)	1	5.68 (0.27–118)
*1/*2	*1/*1	13	9	39.3 (10.6–146)	4	15.79 (3.36–74.11)	5	249 (14.5->999)
			*p* = 0.40 for interaction*		*p* = 0.82 for interaction*		*p* = 0.50 for interaction*	

ADH1B, alcohol dehydrogenase-1B; ALDH2, aldehyde dehydrogenase-2; CI, confidence interval; DIULs, distinct iodine-unstained lesions; MCV, mean corpuscular volume; LGIN, low grade intraepithelial neoplasia; OR, odds ratio; SCC, squamous cell carcinoma

ORs were calculated using "absence" as a control, and were adjusted for age, alcohol drinking, cigarette smoking, BMI, and MCV.

*P* for interaction of ALDH2 genotype by ADH1B genotype on the risk of esophageal DIULs ≥5 mm by a logistic regression analysis. A significant interaction means that the association between ALDH2 genotype and esophageal DIULs ≥5 mm is different by ADH1B*2 allele, and vice versa.

Determinants of multiple DIULs were identified using a multiple logistic regression analysis (Table 6). The presence of the ADH1B*1/*1 genotype increased the risk of multiple DIULs (9.89 [3.50–27.9]), compared with the presence of the ADH1B*2 allele. The presence of the ALDH2*1/*2 genotype increased the OR (18.2 [3.11–106]), compared with the presence of the ALDH2*1/*1 genotype. Other positive determinants of multiple DIULs were a younger age, more smoking, a smaller BMI, and an MCV ≥106 fl.

**Table 6:** Multiple logistic analyses for identifying determinants of multiple DIULs in Japanese alcohol-dependent women.

	Multiple DIULs in the esophagus	
	OR (95% CI)	*P*
Age, per +10 yrs	2.56 (1.58–4.15)	*0*.*0001*
ADH1B *1/*1 vs. *2 carrier	9.89 (3.50–27.92)	*<* .*0001*
ALDH2 *1/*2 vs. *1/*1	3.79 (1.40–10.26)	*0*.*009*
Alcohol consumption, per +22 g ethanol/day	1.10 (0.97–1.25)	*0*.*13*
Smoking, per +10 cigarrettes/day	1.76 (1.21–2.56)	*0*.*003*
Body Mass Index, per -1 kg/m^2^	1.38 (1.14–1.67)	*0*.*001*
MCV, ≥106 fl vs. <106 fl	3.27 (1.24–8.64)	*0*.*017*

ADH1B, alcohol dehydrogenase-1B; ALDH2, aldehyde dehydrogenase-2; CI, confidence interval; DIULs, distinct iodine-unstained lesions; MCV, mean corpuscular volume; OR, odds ratio

Multivariate odds ratios were estimated using a logistic regression model with all the variables entered.

*P* = 0.45 for interaction of ALDH2 genotype by ADH1B genotype on the risk of multiple DIULs by a logistic regression analysis.

## Discussion

In the present study we investigated the determinants of esophageal DIULs ≥5 mm consisting of LGIN and superficial SCC and of esophageal multiple DIULs of any size in Japanese alcohol-dependent women. DIULs ≥5 mm and multiple DIULs were observed in 7.4% (LGIN, 5.5%; SCC, 1.9%) and 6.6% of the patients, respectively. These frequencies were lower than those reported for alcohol-dependent men treated at the same treatment center: 16.2% (LGIN, 7.7% and SCC, 4.6%) and 16.2%, respectively [9]. A younger age and less smoking among the female patients might, at least in part, explain this gender difference.

The inactive ALDH2*1/*2 genotype and the slow-metabolizing ADH1B*1/*1 genotype increased the ORs for DIULs ≥5 mm and multiple DIULs. The magnitude of the effect of ADH1B*1/*1 on the ORs was much higher than that reported in alcohol-dependent men treated at the same treatment center [9]. This effect was larger than that of the ALDH2*1/*2 genotype, and the combination of these two genotypes produced extremely high ORs for DIULs ≥5 mm, LGIN, and SCC. However, the present study was conducted in only 472 women, while the previous study was conducted in 2,115 men [9]. The much lower sample size in the present study might explain the sex difference in the ORs.

Women are likely to have higher blood alcohol levels after consuming the same amount of alcohol, compared with men, because of their smaller liver and muscle sizes, lower water content, and higher lipid content [29]. The average values for typical alcohol consumption and body weight of the presently reported female patients were 12% larger [9] and 16% smaller [30], respectively, than those reported for male patients treated at the same treatment center. Thus, the alcohol consumption per body weight was 33% higher in the female patients than in the male patients. Excessive drinking amplifies the modest effect of the ADH1B*1/*1 genotype on the slow elimination of alcohol [31,32], leading to a much longer exposure to high ethanol concentrations compared with that in ADH1B*2 carriers [32,33]. Furthermore, some ADH1B*1/*1 patients may start to drink again when residual alcohol is still present in their system from their previous drink. This situation is more likely to occur in female patients who drink more and have a smaller lean body weight than male patients. When higher levels of ethanol linger in alcohol-dependent ADH1B*1/*1 carriers, the upper aerodigestive tract is exposed to saliva containing higher levels of acetaldehyde produced by oral microorganisms for longer periods of time [32–34], especially in alcohol-dependent ALDH2*1/*2 carriers [32]. Chronic exposure to acetaldehyde can lead to the formation of acetaldehyde-DNA adducts [35], DNA double-strand breaks causing chromosome rearrangements [36], and the inhibition of enzymes involved in DNA repair [37]. The International Agency for Research on Cancer has defined ‘acetaldehyde associated with alcohol consumption’ as a Group 1 human carcinogen [38].

We have clearly demonstrated that alcohol-dependent men with the ADH1B*1/*1 genotype were younger [39] and had a higher BMI [30] than those with the ADH1B*2 allele. However, age and BMI were not associated with the ADH1B genotype in the present study conducted in women. Since both a younger age and a larger BMI were negative determinants for esophageal DIULs, the lack of these associations may modify the magnitude of the ADH1B*1/*1-associated risk of esophageal DIULs in the female patients. Recent studies have reported that *ADH1B* rs1229984 affects alcohol-independent functions, such as educational attainment and vascular /metabolic conditions, which may also modify the present associations [13,14].

Drinking culture in Japan is much more restrictive for women than for men, and the male-to-female ratio for alcohol-dependence is approximately 10:1 according to the latest nationwide survey [40]. Strong promoting factors are needed to develop alcohol dependence in Japanese women. A previous study reported a high prevalence of psychiatric comorbidities, including eating disorder, depression, panic disorder, and borderline personality disorder, among alcohol-dependent women who visited our Center [41]. The promoting effects of psychiatric comorbidities on alcohol dependence may be one of the reasons for the heavier drinking observed in women.

Among homozygotes and heterozygotes for the inactive ALDH2*2 allele, the blood acetaldehyde levels were approximately 19 and 6 times higher than that among homozygotes for the active ALDH2*1 allele, respectively, in an alcohol challenge test [42]. When individuals with current or former flushing based on their responses to the simple flushing questionnaire were assumed to have inactive ALDH2, the sensitivity and specificity were 90% and 88% for men [26] and 88% and 92% for women [17] in the Japanese general populations. We previously demonstrated that alcohol flushing, when integrated with alcohol consumption, predicts the risk of esophageal SCC as well as the ALDH2 genotype in general populations [17,26]. Mendelian randomization uses genetic polymorphisms that influence alcohol consumption behaviors as surrogates of alcohol exposure [43]. The ALDH2*1/*2 genotype and alcohol flushing are associated with high acetaldehyde exposure after drinking, resulting in a high risk of esophageal SCC. On the other hand, these responses lead to a reluctance to consume alcohol because of the unpleasant effects of the acetaldehyde reaction. Because of the dual action on alcohol consumption behavior and acetaldehyde exposure, the acetaldehyde-associated findings from Mendelian randomization should be integrated with information on alcohol consumption [44].

Since the flushing response is a protective factor against alcohol dependence, alcohol-dependent individuals generally experience weak or no flushing response because of a constitutional or acquired tolerance to acetaldehyde or the presence of the ADH1B*1/*1 genotype, which produces acetaldehyde slowly [45]. The sensitivity and specificity were 74%-75% and 90%-92%, respectively, for alcohol-dependent men at our Center. Alcohol-dependent men with current or former flushing had an OR of 3.31 for esophageal SCC in a case-control study, with a hazard ratio of 2.36 for upper aerodigestive tract SCC in a follow-up study. However, the corresponding risks for the ALDH2*1/*2 genotype *per se* were even higher because of the 74%-75% sensitivity of the flushing as a proxy for the inactive ALDH2 [45]. In the present study conducted in women, the sensitivity was even lower: 50.0% overall and only 31.8% in the ALDH2*1/*2 plus ADH1B*1/*1 carriers. Thus, alcohol flushing cannot predict the high prevalence of ALDH2*1/*2 among patients with DIULs ≥5 mm. Alcohol flushing may play a more crucial role in the prevention of developing alcohol dependence in women than in men in Japanese drinking culture. Women may tend to view the facial flushing response as shameful and a cosmetic problem under the restrictive female drinking culture in Japan. Alcohol-induced flushing is triggered by a steep initial rise in blood acetaldehyde after drinking and does not tend to occur in persons with the slow-acetaldehyde-producing ADH1B*1/*1 genotype [45]. Persons who lack an alcohol flushing response are probably more vulnerable to alcohol dependence among Japanese women with ALDH2*1/*2. The strong association between the ADH1B*1/*1 genotype and esophageal DIULs observed in this study may be partly attributable to the weak alcohol flushing in alcohol-dependent women with the ADH1B*1/*1 genotype.

Macrocytosis of MCV ≥106 fl and a lower BMI increased the ORs for both DIULs ≥5 mm and multiple DIULs in the female patients. These findings were consistent with the results that both an MCV ≥106 fl and a low BMI were associated with a risk of esophageal SCC in a case-control study [24] and a follow-up study [12] in alcohol-dependent men performed at the same treatment center. A low BMI increased the risk of newly diagnosed high-grade dysplasia or SCC in the esophagus in a Japanese cohort study [46]. The low BMI observed in alcohol-dependent individuals is mainly attributable to their poor dietary habits, leading to vitamin deficiencies and suppressed immune function. Evidence indicates that the MCV in inactive ALDH2 drinkers is increased by exposure to acetaldehyde [24,47]. Excessive drinking, acetaldehyde exposure, smoking, a low BMI, and folate deficiency are associated with both an increased MCV and an increased risk of aerodigestive tract cancer [12,24,47].

The presence of LGIN in DIULs ≥5 mm was correlated with the presence of the ALDH2*1/*2 genotype and the ADH1B*1/*1 genotype in alcohol-dependent men treated at our treatment center [9] and was associated with a very high risk of the future development of SCC in the upper autodigestive tract [25]. The 5-year cumulative rate of SCC detection in the upper aerodigestive tract was more than 30% in those with dyaplastic DIULs ≥5 mm, as opposed to 4% in those without DIULs ≥5 mm, and its hazard ratio was 5.88 [25]. The presence of multiple esophageal DIULs is another marker of multicentric cancerization in the upper aerodigestive tract [8,10,48–52] and is associated with alcohol drinking, smoking, the ALDH2*1/*2 genotype, and the ADH1B*1/*1 genotype [8–10]. Furthermore, it has been demonstrated to be a strong predictor of the development of metachronous SCC in the upper aerodigestive tract after endoscopic mucosectomy for esophageal SCC [48–52]. In the present study, 45.7% of the DIULs ≥5 mm were accompanied by the presence of multiple DIULs, and both types of DIULs were positively associated with the ALDH2*1/*2 genotype, the ADH1B*1/*1 genotype, a low BMI, and an MCV ≥106 fl. The DIULs in the alcohol-dependent women may indicate a high-risk background for future multicentric cancerization in the upper aerodigestive tract, and this issue will be an important theme of future research.

Our study had several potential limitations. The small sample size in this study limited the risk assessment, and the neoplastic progression from large DUULs or multiple DIULs to SCC was not directly evaluated. Such evaluations require longitudinal follow-up screening examinations in larger studies. We also did not have any data regarding the ethanol and acetaldehyde levels in the female patients. Ethanol and acetaldehyde measurements of blood and saliva obtained while the patients were intoxicated might have clarified whether any gender differences in exposure levels to ethanol and acetaldehyde exist according to the ADH1B and ALDH2 genotypes. We did not observe any effects of alcohol consumption during the preceding year on the DIULs. These findings may be related to the homogeneity of the study population, since all the patients had extremely high alcohol consumption. Since long-term exposure is required to develop neoplasia, it would be more relevant to study the effects of alcohol consumption over a longer period.

In conclusion, alcohol-dependent women and men share several common risk factors of esophageal neoplasia, as diagnosed by the presence of DIULs ≥5 mm, and multiple DIULs, but with considerably different magnitudes. These factors include the slow-metabolizing ADH1B*1/*1 genotype, the inactive heterozygous ALDH2*1/*2 genotype, a low BMI, and a large MCV.
